# Multifunctional Polymer Composite Materials, 2nd Edition

**DOI:** 10.3390/polym17212847

**Published:** 2025-10-25

**Authors:** Md Najib Alam, Vineet Kumar

**Affiliations:** School of Mechanical Engineering, Yeungnam University, 280, Daehak-ro, Gyeongsan 38541, Republic of Korea; mdnajib.alam3@gmail.com

## 1. Introduction

Polymer composite materials have emerged as a key class of advanced materials with robust properties. These features are due to their ability to combine the inherent flexibility, lightweight nature, and processability of polymers [1]. They also exhibit superior mechanical, electrical, or thermal properties inherited from reinforcing fillers. Traditionally, composites were designed for a single purpose, such as enhancing strength or reducing weight. However, modern applications in electronics, energy, aerospace, biomedical devices, and smart textiles demand multifunctionality. Therefore, these polymer composite materials can perform multiple roles simultaneously, giving rise to multifunctional materials. Multifunctional polymer composites integrate different types of fillers into a polymer matrix [2]. These fillers are carbon nanotubes, graphene, boron nitride, ceramics, or metallic nanoparticles. They also impart additional features like electrical conductivity, thermal management, self-healing, piezoelectricity, or sensing capabilities. Moreover, they maintain essential mechanical robustness and flexibility. The synergy between the polymer matrix and multifunctional fillers is critical for these composites. They act not only as structural materials but also as active components in energy-harvesting devices [3]. These devices are useful in flexible electronics, sensors, actuators, and self-powered systems. Furthermore, multifunctionality in polymer composites extends beyond property enhancement; it enables adaptability to complex environments. For instance, a single composite material can simultaneously provide strength, conductivity, and environmental resistance.

However, for multifunctional composites, the system can provide multiple functional aspects in terms of enhanced multiple properties for various applications in a system. This integration improves efficiency, reduces weight, and enhances the scalability of next-generation technologies. In summary, multifunctional polymer composite materials represent a transformative direction in materials science, bridging mechanical performance with advanced functional capabilities. Their versatility positions them as crucial enablers of smart, sustainable, and miniaturized technologies for future applications [4]. In an overview of these concepts, the present issue explores an in-depth investigation of multifunctional aspects of different polymer composites. Moreover, the papers published in this issue will provide an in-depth study on various multifunctional aspects, both at the academic and industrial scales. Here, the following section will discuss the literature collected on the topic across various domains. Overall, the target application of this Special Issue includes energy harvesting and sensing, both of which are described in this issue in the form of detailed research papers.

## 2. Overview of Published Articles

Kumar et al. [5] develops piezoelectric nano-generators based on silicone rubber, titanium oxide (TiO_2_), and molybdenum disulfide (MoS_2_). These composites are fabricated by solution-mixing and -molding process. The results are interesting, and robust mechanical and electro-mechanical properties are described. Finally, piezoelectric co-efficient and power density are studied and reported. For example, the piezoelectric coefficients of the PENGs were 40 pC/N (TiO_2_), 112 pC/N (MoS_2_), and 160 pC/N (hybrid filler). These improved properties are beneficial for various applications like in self-powered portable electronics. Similarly, Yoldaş et al. [6] studied damages at filler–rubber interfaces in adhesively bonded polymer composite systems. The author discusses the influence of filler orientation, twill-woven filler particles according to ASTM D5868-01 standard. Then, the properties are studied through three-point bending tests according to the ASTM D790 standard. Finally, diffusion and retention behavior was investigated through Fick’s law, and their prospective applications in seawater were evaluated. In another study by Shahgodari et al. [7], the results propose the examination of the recovery of total ammoniacal nitrogen (TAN). This TAN was recovered using thin film based on polymer composites and was useful for various applications. The results were interesting and show that the rejection of TAN was influenced significantly by the type of membrane, pH, and presence of ammonium salt. In a study by De la Cruz et al. [8], the tunable fiber-reinforced shape memory polymer composites were presented. As known, shape memory polymer composites systems are often termed as intelligent materials. These materials are capable of tuning shape through actuation and promising stimuli-based response systems. Here, the effects of adding PEG-600 on mechanical and thermal properties are described. Results show that the tensile strength of final composites are 233.59 MPa, and the Young’s modulus is as high as 14.081 GPa. These improved properties found the use of PEG as a plasticization agent promising by tuning the structural performances of the composites. In another interesting study by De la Cruz et al. [9], the authors demonstrate the development of a sequential antenna through fiber-reinforced shape memory polymer composite systems. The mechanisms show that thermal properties like glass transition can be tuned via the addition of PEG-600 and are useful for sequential actuations. The results also signify that samples recover fully into their original shapes within 2 min of removing mechanical strain. However, further simulation is required to access and validate their final performances for particular applications. Finally, Maqsood et al. [10] present interesting results on composites based on stainless steel fillers fabricated through an injection-molding process. After fabrication, their microstructure, mechanical, thermal, and wear behaviors are reported. The study especially emphasizes structure–property relationships, such as the effect of the filler morphology on various properties. For example, the modulus of neat PA12 was 0.95 GPa, while the tensile strength was 35.04 MPa. Overall, the stainless steel enhances stiffness and wear resistance, resulting in balanced properties, and can be tuned based on requirement of application of interest.

## 3. Summary and Future Outlook

Multifunctional polymer composite materials have emerged as a transformative class of engineered systems recently. They are integrated in terms of mechanical robustness, electrical responsiveness, and chemical adaptability within a single platform. These features are achieved by combining flexible polymer matrices with functional fillers. These fillers include carbon nanotubes (CNTs), graphene, hollow carbon microspheres (HCMs), boron nitride, or metal oxides [11]. Moreover, these composites exhibit tailored properties, including enhanced strength, electrical conductivity, thermal stability, self-healing, and energy-harvesting capability. The synergy between polymer elasticity and filler functionality enables these composites to operate as sensors, nanogenerators, and flexible electronics. The underlying mechanisms involve interfacial polarization, filler percolation, and mechanical–electrical coupling, which govern their performance [12]. Advanced processing techniques like roll-to-roll fabrication, 3D printing, and solution casting have further facilitated scalable and uniform production of these materials. Recent studies have demonstrated that optimizing the filler content, morphology, and surface modification enhances energy density, power output, and signal stability. Importantly, the integration of AI-driven material design, data analytics, and machine learning-assisted optimization is accelerating the discovery of new composite formulations with a superior multifunctional performance [13]. Overall, by uniting the properties, these materials hold the potential to redefine the landscape of next-generation applications. These applications are soft electronics, wearable energy systems, and smart human–machine interfaces. Thus, the interdisciplinary research provides a vital means for unlocking their full potential in practical, scalable, and environmentally responsible applications.

Looking ahead, the next generation of multifunctional polymer composites is expected to evolve further. These aspects are toward intelligent, adaptive, and sustainable systems with enhanced performance and functionality. For example, the application of artificial intelligence (AI) and machine learning (ML) will enable rapid prediction of property–composition–process relationships [14]. Moreover, the future composites will be engineered to respond simultaneously to multiple external stimuli. These stimuli are pressure, temperature, strain, and humidity. Similarly, they enable real-time sensing, self-regulation, and adaptive energy conversion in smart electronic systems [15]. Moreover, the development of biodegradable, recyclable, and bio-based polymer matrices will address environmental concerns. Therefore, the sustainable synthesis routes and solvent-free processing will support large-scale, eco-friendly production. In addition, improved filler–matrix interfacial engineering, hybrid crosslinking strategies, and self-healing chemistries will be developed and need further attention. This will ensure fatigue resistance and voltage stability during prolonged cyclic operation [16]. Achieving a breakthrough in this area is critical for wearable and industrial applications. Similarly, multifunctional polymer composites will be integrated into flexible circuits, soft robotics, and IoT-connected devices. These devices will be fabricated based on self-powered electronic skins, health-monitoring patches, and haptic feedback systems. Finally, emerging fabrication methods, such as additive manufacturing and layer-by-layer assembly, will enable hierarchical architectures [17]. These will be preferred due to their ability to control filler alignment, resulting in tunable anisotropic properties and enhanced electromechanical coupling.
